# Long-term visual quality after small incision lenticule extraction (SMILE) and laser assisted subepithelial keratomileusis (LASEK) for low myopia

**DOI:** 10.1186/s12886-022-02568-8

**Published:** 2022-08-18

**Authors:** Mengjun Fu, Meiyan Li, Ruoyan Wei, Chuanwei Zhang, Yangyi Huang, Lingling Niu, Xiaoying Wang, Haorun Zhang, Xingtao Zhou

**Affiliations:** 1 Department of Ophthalmology, Weifang Eye Hospital, No.139 Xingfu Road, Kuiwen District, Weifang, 261000 Shandong People’s Republic of China; 2 State Key Laboratory of Ophthalmology, Optometry and Visual Science, Eye Hospital of Wenzhou Medical University, Wenzhou, Zhejiang China; 3 National Clinical Research Center for Ocular Diseases, Eye Hospital of Wenzhou Medical University, Wenzhou, Zhejiang China; 4 Eye Institute and Department of Ophthalmology, Eye & ENT Hospital, Fudan University, Shanghai, 200031 China; 5 NHC Key Laboratory of Myopia, Key Laboratory of Myopia, (Fudan University), Chinese Academy of Medical Sciences, Shanghai, 200031 China; 6 Shanghai Research Center of Ophthalmology and Optometry, Shanghai, China; 7 Affiliated Hospital of Nanjing University of Chinese Medicine, Nanjing, China

**Keywords:** Small incision lenticule extraction (SMILE), Laser assisted subepithelial keratomileusis (LASEK), Myopia, Visual quality

## Abstract

**Background:**

Few studies have reported the visual outcomes of small-incision lenticule extraction (SMILE) and laser-assisted subepithelial keratomileusis (LASEK) for myopia correction. This study aims to compare the visual quality and corneal wavefront aberrations after SMILE and LASEK for low-myopia correction.

**Methods:**

In this prospective study, we included 29 eyes of 29 patients who received SMILE and 23 eyes of 23 patients who received LASEK between June 2018 and January 2019. The following measurements were assessed: uncorrected (UDVA) and corrected (CDVA) distance visual acuity, manifest refraction, corneal wavefront aberrations, and subjective visual quality. All patients were followed up for two years.

**Results:**

All procedures were uneventful. An efficacy index of 1.19 ± 0.17 was established in the SMILE group and 1.23 ± 0.20 in the LASEK group. No eyes lost more than two lines of CDVA. We found that 93% (27/29) of the treated eyes in the SMILE group and 91% (21/23) in the LASEK group had spherical equivalent (SE) within ± 0.25D. The increases in the total corneal spherical aberration and the corneal front spherical aberration were lower in the SMILE group than in the LASEK group (*P* < 0.01). In contrast, the increases in the total corneal vertical coma and the corneal front vertical coma in the SMILE group were greater than those in the LASEK group (*P* < 0.01).

**Conclusion:**

Both SMILE and LASEK have good safety, stability, and patient-reported satisfaction for low myopia. SMILE induced less corneal spherical aberration but greater vertical coma than LASEK.

## Background

Corneal refractive surgeries have experienced rapid development in the past few decades. Procedures, such as photorefractive keratectomy (PRK), laser-assisted subepithelial keratomileusis (LASEK), laser in situ keratomileusis (LASIK), femtosecond-laser-assisted LASIK, and small-incision lenticule extraction (SMILE), have shown favorable results in terms of efficacy, safety, stability, and predictability in the correction of myopia. LASEK has gained worldwide acceptance for low-myopia correction [1–5]. First reported in 2011 by Sekundo and Shah [6, 7], SMILE is a novel all-in-one procedure without the creation of a corneal flap. So far, SMILE has had good results in the correction of low myopia in several reported studies [8–10]. However, to the best of our knowledge, no evidence exists of comparisons of subjective visual quality between SMILE and LASEK applied for low-myopia correction.

In the present study, we aimed to compare the 2-year visual outcomes, objective and subjective visual quality of SMILE and LASEK applied for low-myopia and myopic astigmatism correction.

## Inclusion and exclusion criteria

The study was conducted in compliance with the principles of the Helsinki Declaration and was approved by the Ethics Committee of Fudan University's EENT Hospital Review Board. Each patient signed an informed consent form after a detailed explanation of the risks and benefits of the study.

The following inclusion criteria were applied: (1) Age between 20 and 40 years; (2) Spherical error between -3.00D and -0.50D, cylinder up to 1.50D, corrected distance visual acuity (CDVA) less than or equal to 0.0 (log MAR); (3) Stable refractive error (annual change of refractive error less than 0.50D in the past two years); (4) Thickness of the residual corneal stromal bed greater than 280 μm; (5) Patients who wore contact lenses were required to discontinue wearing soft ones for one week and hard ones for more than two weeks. Exclusion criteria: (1) Suspicious keratoconus; (2) A history of eye trauma or eye surgery; (3) Other eye diseases or systemic diseases affecting the eyes.

In this study, we included a total number of 52 patients (52 eyes) with low myopia who underwent SMILE or LASEK surgery between June 2018 and January 2019 at the Fudan University Eye and ENT Hospital (Shanghai, China). The SMILE group was composed of 29 patients (29 eyes; male: female ratio, 8:21; age: 26.8 ± 5.2 years), and 23 patients (23 eyes) constituted the LASEK group (male: female ratio, 11:12; age: 29.3 ± 5.1 years). All patients were enrolled with one eye, and if both eyes met the criteria, the right eye was selected (Table 1).

**Table 1:** Patient profiles

	SMILE group (*n* = 29 eyes)		LASEK group (*n* = 23 eyes)		*P*-values	
	Mean ± SD	Range	Mean ± SD	Range	*t*	*P*
Age (years)	26.79 ± 5.19	21, 40	29.34 ± 5.13	20, 40	1.77	0.08
Gender(male/female)	21/8		11/12		-	0.10
Sphere (D)	-2.11 ± 0.69	-3.00, -1.00	-2.32 ± 0.69	-3.0, -0.75	-1.12	0.27
Cylinder (D)	0.70 ± 0.39	0, 1.5	0.62 ± 0.42	0, 1.5	-0.70	0.49
SE (D)	-2.54 ± 0.72	-3.75, -1.13	-2.58 ± 0.83	-3.88, -1.00	-0.19	0.85
CCT (μm)	547.86 ± 26.92	500, 598	516.09 ± 34.89	460, 578	-3.71	< 0.01^*^
IOP (mmHg)	15.22 ± 2.14	11.3, 19.3	14.20 ± 2.31	11, 18.2	-1.64	0.11
Ablation depth (μm)	70.48 ± 12.30	52, 98	55.65 ± 15.50	28, 81	-3.85	< 0.01 ^*^
AL (mm)	24.97 ± 0.72	23.23, 27.01	24.82 ± 0.66	23.31, 26.32	-0.79	0.44

*SMILE* small incision lenticule extraction, *LASEK* laser-assisted sub-epithelial keratomileusis, *SE* spherical equivalent, *CCT* central corneal thickness, *IOP* intraocular pressure, *D* diopters, *AL* axial length

^*^ *p* < 0.05

### Main refractive and biometric measures

Before and post-operatively, patients underwent routine ophthalmic examinations, including (1) Anterior segment slit-lamp and fundus examination; (2) Visual acuity. A standard logarithmic acuity chart was used for the visual acuity examination. Uncorrected distance visual acuity (UDVA) and CDVA were included, and manifest refraction was performed; (3) Ocular axial length (AL) (Humphrey IOL Master, Carl Zeiss Meditec, Germany) and intraocular pressure (IOP); (4) Corneal topography (Pentacam HR, Type 70,900; Oculus Optikgerate GmbH, Wetzlar, Germany); (5) Wavefront corneal aberrations were calculated by Zernike Analysis on Pentacam (Zernike Order: 6, Refractive: Cornea: 1.376, Aqueous:1.336).RMS HOAs, spherical aberrations, coma, and trefoil were selected from the corneal central zone diameter of 6 mm. All patients were followed up for two years.

### Subjective visual quality

Questionnaires were used to obtain information from patients about their subjective visual quality. The common visual complaints after refractive surgery were selected, including glare, halo, starburst, hazy vision, blurred vision, and vision fluctuation. Vision fluctuation refers to a symptom of changes in visual acuity over time. The first five items were provided with corresponding pictures to reduce the possibility of an irrelevant answer. In addition, the patients were asked how much their visual quality improved post-operatively, about their overall satisfaction with the procedure, and whether they would like to recommend the procedure to others.

### Surgical techniques

SMILE was performed through the following procedure steps: The Visumax femtosecond laser system (Carl Zeiss Meditec AG, Jena, Germany) with a frequency of 500 kHz and the expert mode was used. The following settings were applied: pulse energy of 130 nJ, the thickness of the cornea cap of 120 μm, optical zone diameter of 6.5 to 6.8 mm, and a base thickness of 10 μm. The upper side incision was set at 90° (12 o'clock), and the width was 2.0 mm. Point spacing of 2.5 μm was used for lenticule cutting and cap cutting, and point spacing of 2.0 μm was adopted for lenticule side-cutting and cap-side cutting. Then, the lenticule was separated and removed.

LASEK was performed in the following steps. The Triple-A model of the Carl Zeiss MEL 90 excimer laser system (Carl Zeiss Meditec AG, Jena, Germany) was used. First, the corneal epithelium was soaked in 20% alcohol for 12 s, and the upper corneal epithelium flap was separated by the corneal epithelium shovel. The corneal stromal bed was exposed, and an excimer laser was applied, with a laser frequency of 500 Hz and an optical zoon diameter of 6.5 mm. The corneal stromal bed was rinsed with Ringer's fluid, the upper corneal flap was repositioned, and a corneal bandage lens was placed.

### Postoperative medication

Eye drops were used in the SMILE group as follows: 0.5% levofloxacin (Cravit; Santen, Osaka, Japan), four times daily for seven days; 0.1% fluorometholone (Fluorometholone; Santen) eight times daily and tapered to one time daily for over 24 days; and artificial tears (Hyalein, 0.1% hyaluronic acid, Santen) four times daily for one month.

Eye drops were used in the LASEK group as follows: 0.5% levofloxacin (Cravit; Santen) four times daily for seven days. The contact lenses were removed 5–7 days post-operatively. 0.1% fluorometholone (Fluorometholone; Santen) eight times daily for one week, seven times daily for one week, and then tapered to one time daily for over 14 weeks. Artificial tears (Hyalein, 0.1% hyaluronic acid; Santen) were applied four times daily for three months.

### Statistical analysis

Statistical analysis was performed using R version 3.6.2 (R Project for Statistical Computing, http://cran.rproject.org). Continuous variables were expressed as mean ± standard deviation (SD), whereas categorical variables were represented as frequency and percent. Shapiro–Wilk test was executed to examine the data for normal distribution. For normally distributed variables, independent *t*-tests were performed, whereas the Wilcoxon test was applied for variables that were not normally distributed. The chi-square test was used to assess the statistical significance of differences in percentages. *P*-values less than 0.05 were considered to indicate statistically significant differences.

## Results

### Visual outcomes

**Fig. 1:**
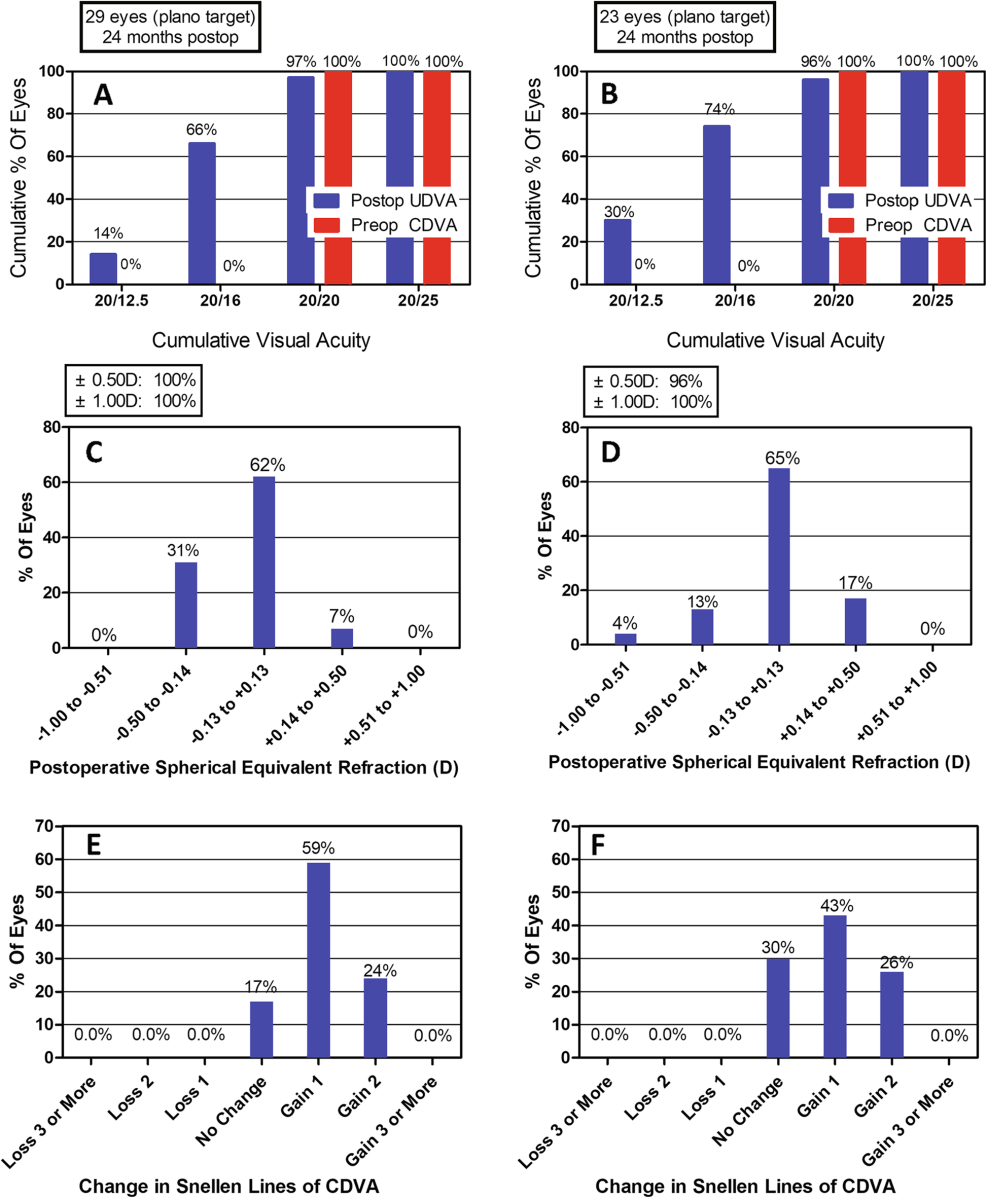
**A**-**B** Postoperative uncorrected distance visual acuity (UDVA) and preoperative corrected distance visual acuity (CDVA) between the SMILE (**A**) and LASEK (**B**) groups. **C**-**D** Spherical equivalent in the SMILE (**C**) group and LASEK (**D**) groups. **E**–**F** Changes in the lines of the CDVA in the SMILE (**E**) and LASEK (**F**) groups. Plano: zero diopter; Postop: postoperative; Preop: preoperative; SMILE: small incision lenticule extraction; LASEK: Laser-assisted subepithelial keratomileusis

All surgical operations were uneventful without any complications such as infection (Table 2). The 2-year efficacy index (postoperative UDVA/preoperative CDVA) in the SMILE and LASEK groups were 1.19 ± 0.17 and 1.23 ± 0.20, respectively (*P* = 0.42). 97% (28/29) of SMILE-treated eyes and 96% (22/23) of LASEK-treated eyes had a UDVA of 0 or better (log MAR). Eyes in both groups had a UDVA of 0.1 or better (log MAR). 97% (28/29) of SMILE-treated eyes (Fig. 1A) and 96% (22/23) of LASEK-treated eyes (Fig. 1B) had a UDVA equal to or better than preoperative CDVA. 93% (27/29) of SMILE-treated eyes and 91% (21/23) of LASEK-treated eyes had spherical equivalent (SE) within ± 0.25D, 100% (29/29) of SMILE-treated eyes (Fig. 1C) and 96% (22/23) of LASEK-treated eyes ( Fig. 1D) within ± 0.50D, and all the eyes within ± 1.00D.

**Table 2:** Parameters of the two groups

	SMILE		LASEK		*P*-values	
	Mean ± SD	Range	Mean ± SD	Range	t	P
UDVA	-0.08 ± 0.07	-0.20, 0.05	-0.10 ± 0.08	-0.20, 0.05	-1.09	0.28
CDVA	-0.11 ± 0.07	-0.20, 0.05	-0.12 ± 0.07	-0.20, 0.00	-0.81	0.42
Efficacy index	1.19 ± 0.17	0.90, 1.50	1.23 ± 0.20	0.90, 1.50	0.81	0.42
Safety index	1.14 ± 0.17	1.00, 1.50	1.28 ± 0.18	1.00, 1.50	0.84	0.41
Residual Sphere (D)	-0.02 ± 0.20	-0.50, 0.50	0.15 ± 0.18	0.00, 0.50	3.17	0.00
Residual cylinder (D)	0.11 ± 0.18	0.00, 0.50	0.16 ± 0.22	0.00, 0.75	0.91	0.37
Residual SE (D)	-0.07 ± 0.18	-0.50, 0.25	-0.02 ± 0.21	-0.63, 0.38	1.04	0.30
CCT (μm)	485.69 ± 28.31	435, 542	466.43 ± 41.56	400, 546	-1.98	0.05
IOP (μm)	11.20 ± 1.83	7.40, 14.80	11.17 ± 1.55	9.20, 16.00	-0.07	0.95
AL (mm)	24.92 ± 0.77	23.12, 26.82	24.73 ± 0.71	23.13, 26.14	-0.92	0.36

*SMILE* small incision lenticule extraction, *LASEK* laser-assisted subepithelial keratomileusis, *UDVA* uncorrected distance visual acuity, *CDVA* corrected distance visual acuity, *SE* spherical equivalent, *D* diopters, *CCT* central corneal thickness, *IOP* intraocular pressure, *AL* axial length

The safety index (postoperative CDVA/preoperative CDVA) of the SMILE group and LASEK group were 1.24 ± 0.17 and 1.28 ± 1.18, respectively (*P* = 0.41). 59% (17/29) of SMILE-treated eyes and 43% (16/23) of LASEK-treated eyes gained a one-line improvement in CDVA. 24% (7/29) of SMILE-treated eyes (Fig. 1E) and 26% (6/23) of LASEK-treated eyes (Fig. 1F) gained two-line. In either group, no eyes lost two or more lines of CDVA.

**Fig. 2:**
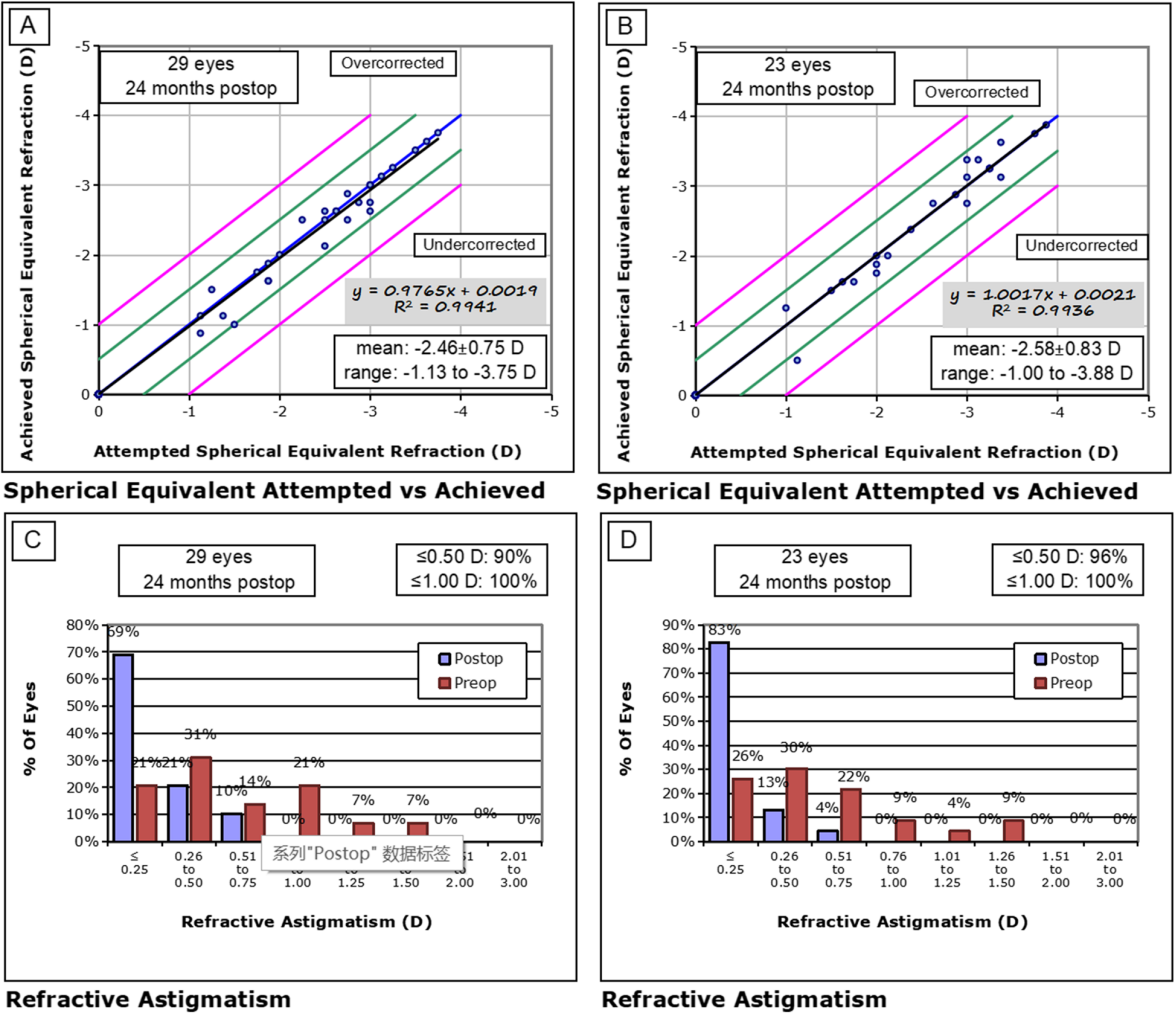
**A-B** Spherical equivalent (SE) attempted versus achieved in the SMILE (**A**) and LASEK (**B**) groups. **C-D** Postoperative refractive astigmatism and preoperative refractive astigmatism in the SMILE (**C**) and LASEK (**D**) groups. Postop: postoperative; Preop: preoperative. SMILE: small incision lenticule extraction; LASEK: Laser-assisted subepithelial keratomileusis

The predictability is represented in Fig. 2A and Fig. 2B by scatter plots of the difference between the target SE and the achieved SE of the SMILE and LASEK groups, respectively.

**Fig. 3:**
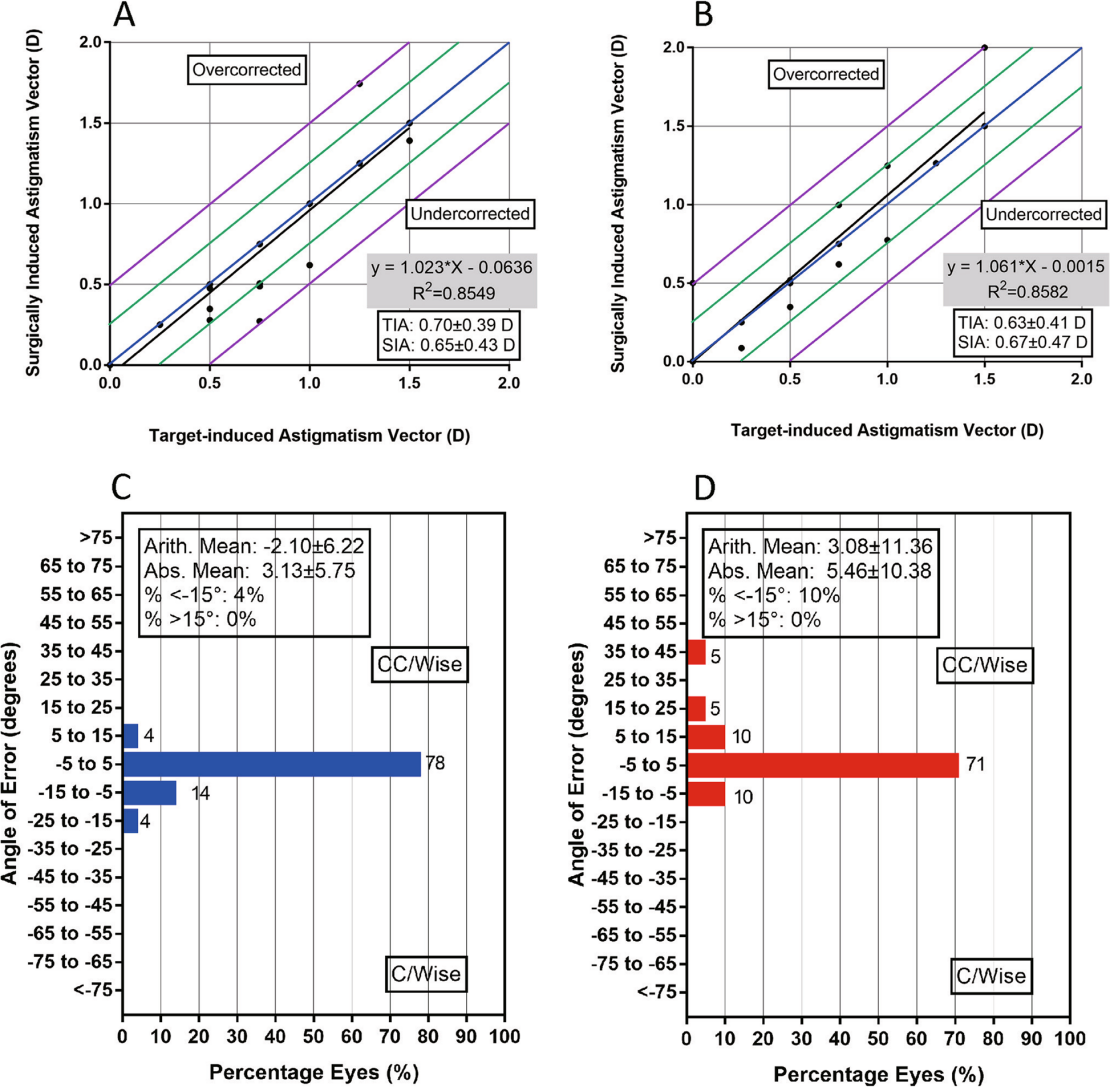
Scatter plot of the achieved versus attempted correction of astigmatic vectors after SMILE (**A**) and LASEK (**B**). Percentage of eyes according to the angle of error (degrees) after SMILE (**C**) and LASEK (**D**). SMILE: small incision lenticule extraction; LASEK: Laser-assisted subepithelial keratomileusis

In terms of stability, the postoperative SE of the SMILE and LASEK groups were -0.07 ± 0.18D and -0.02 ± 0.21D, correspondingly. Postoperative astigmatism in the SMILE (Fig. 2C) and LASEK (Fig. 2D) groups was 0.11 ± 0.18D and 0.11 ± 0.22D, respectively. A scatter plot of the target-induced astigmatism versus the surgically induced astigmatism is shown in Fig. 3A-B. Percentage of eyes according to the angle of error (degrees) is presented in Fig. 3C-D.

### Corneal wavefront aberrations (6 mm)

**Fig. 4:**
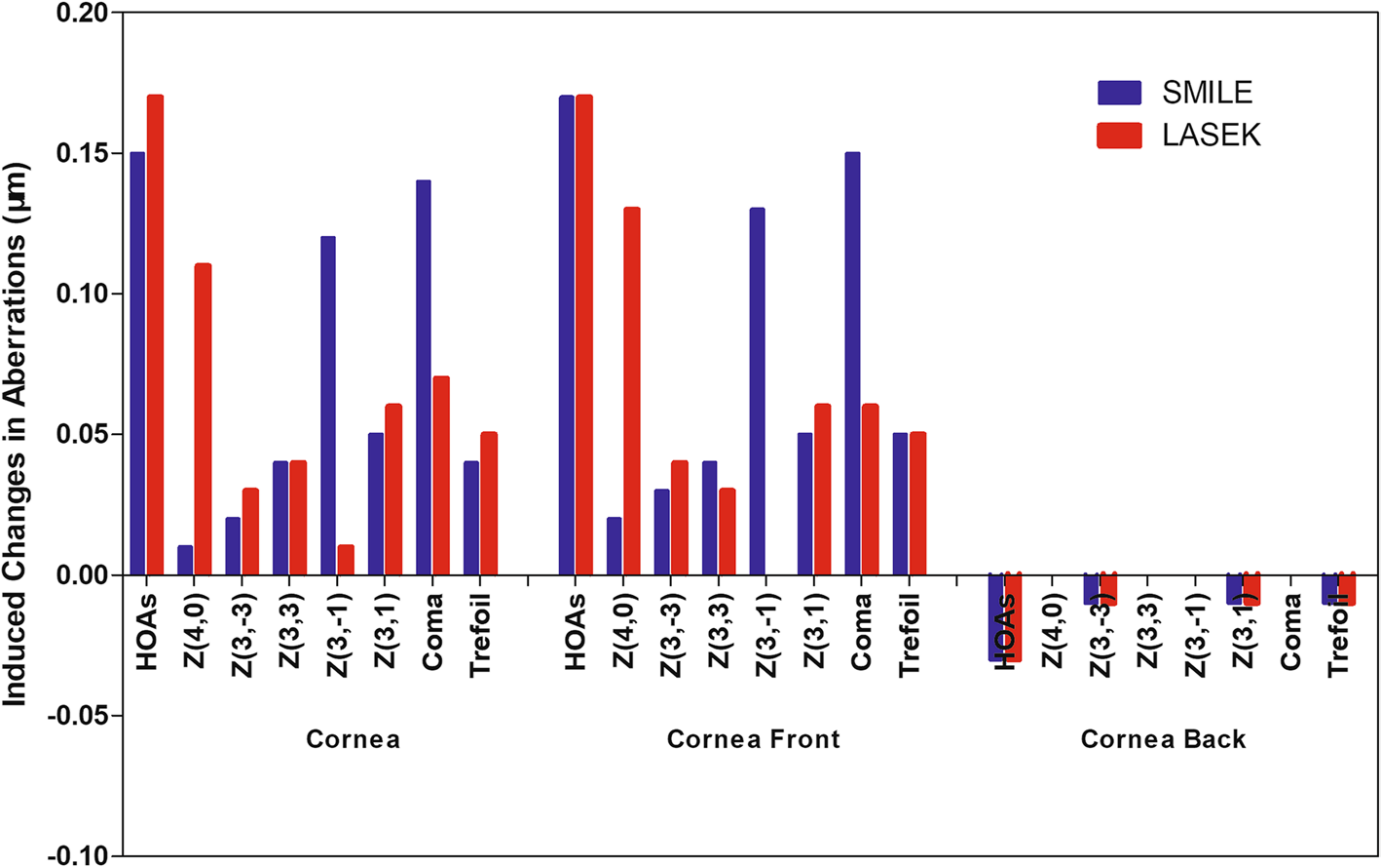
Induced changes in wavefront aberrations (6.0-mm analysis corneal diameter) in the SMILE and LASEK groups. Postop: postoperative; RMS: root mean square; HOAs: higher-order aberrations; Z4,0: spherical aberration; Z3, -3: oblique trefoil; Z3, -1: vertical coma; Z3, 1: horizontal coma; Z3, 3: horizontal trefoil. SMILE: small incision lenticule extraction; LASEK: Laser-assisted subepithelial keratomileusis

Total corneal HOAs and corneal front surface HOAs were increased in both groups postoperatively, but the increases in HOAs were not statistically significant (*P*-values of 1.00 and 0.68, respectively). The postoperative increase of total corneal spherical aberration and corneal front surface spherical aberration in the SMILE group was less than that in the LASEK group (*P* < 0.01), and the increase of total corneal vertical coma and corneal front surface vertical coma were greater than that of LASEK (*P* < 0.01) (Fig. 4).

### Postoperative subjective visual quality and patient satisfaction

**Fig. 5:**
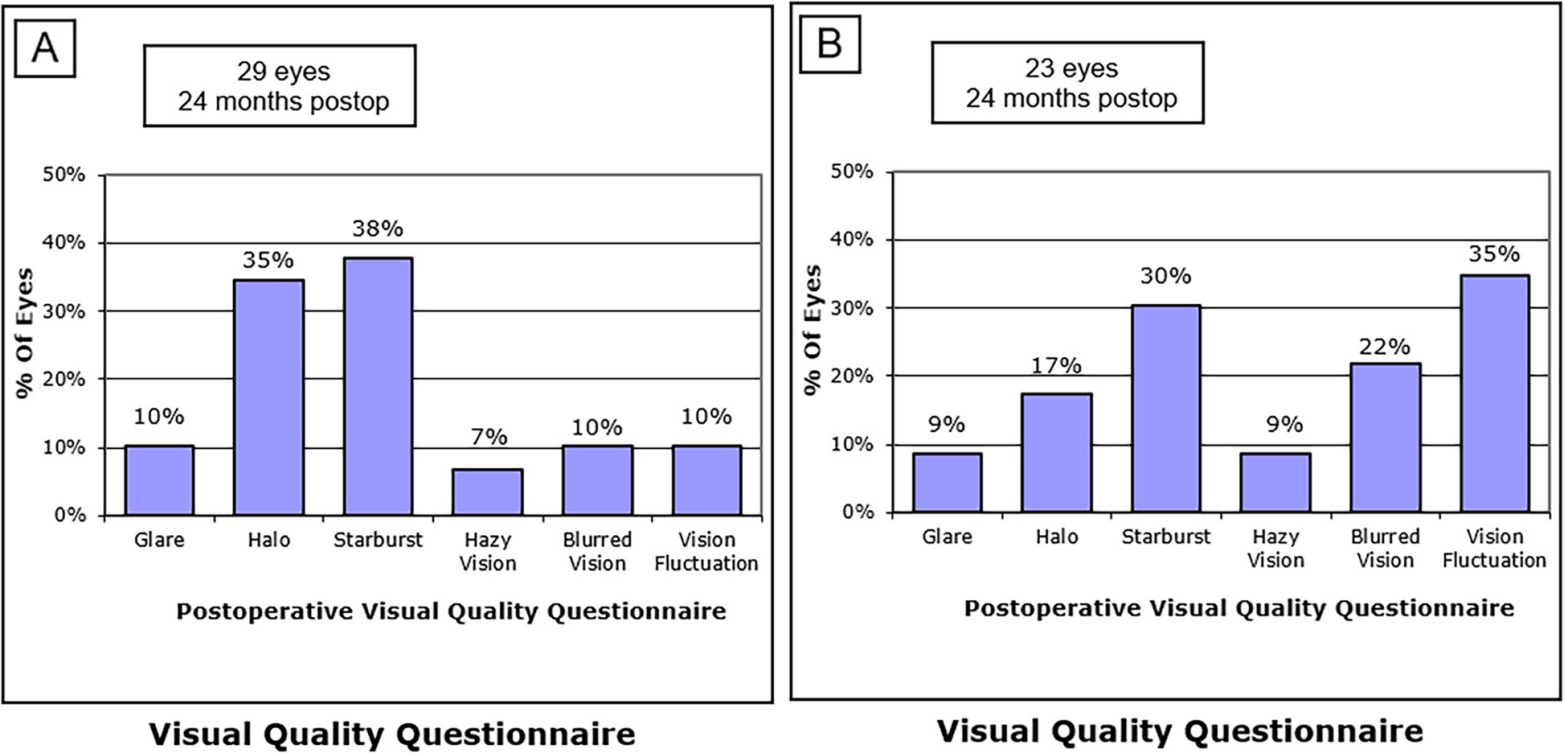
Visual complaints in the SMILE (**A**) and LASEK (**B**) groups. Postop: postoperative; SMILE: small incision lenticule extraction; LASEK: Laser-assisted subepithelial keratomileusis

The most common postoperative visual complaints in both groups were starburst, halo, and vision fluctuation (Table 3). We observed starburst in 38% (11/29) of the SMILE-treated eyes and 30% (7/23) of the LASEK-treated eyes. Additionally, we established that 35% (10/29) of the SMILE-treated eyes and 17% (4/23) of the LASEK-treated eyes experienced halo. Only 10% (3/29) of the SMILE-treated eyes and 22% (5/23) of the LASEK-treated eyes suffered from blurred vision, and 10% (3/29) of the SMILE-treated eyes and 35% (8/23) of the LASEK-treated eyes experienced visual fluctuation. Most of the visual symptoms reported were mild, without daily-life disturbance. It is noteworthy that 93% (27/29) of the SMILE group patients and 87% (20/23) of the LASEK group ones were satisfied with the treatment. Moreover, 97% (28/29) of the SMILE-treated patients and 96% (22/23) of the LASEK-treated ones felt that their visual quality was significantly improved as compared with the preoperative levels. Notably, 86% (25/29) of the patients in the SMILE group (Fig. 5A) and 91% (21/23) of patients in the LASEK group (Fig. 5B) were willing to recommend surgery to myopic patients with similar conditions.

**Table 3:** Percentage of postoperative subjective visual quality and satisfaction questionnaire

	SMILE (*n* = 29 eyes)		LASEK (*n* = 23 eyes)	
	n	*P* (%)	n	*P* (%)
Glare	3	10	2	9
Halo	10	35	4	17
Starburst	11	38	7	30
Hazy Vision	2	7	2	9
Blurred Vision	3	10	5	22
Vision Fluctuation	3	10	8	35
Very satisfied	27	93	20	87
Significantly improved quality of life	28	97	22	96
Would like to recommend	25	86	21	91

*SMILE* small incision lenticule extraction, *LASEK* laser-assisted subepithelial keratomileusis

## Discussion

SMILE has been widely accepted for the correction of myopia less than -12.0 D; LASEK has also been shown to have good visual outcomes in the correction of low myopia [2, 9, 11]. In the present study, we compared for the first time the subjective visual quality and corneal aberrations obtained by the two procedures applied for low myopia correction.

Both SMILE and LASEK showed good safety, efficacy, and predictability in the correction of low myopia, which was consistent with the results of previous studies [9, 10]. Reinstein et al. [10] reported that after the application of SMILE (mean SE: -2.61 ±—0.54 D) for low-myopia with 1-year follow-up, 96% of the patients had UDVA of 20/20 or better and UDVA of 20/25 or better with mean residual SE of -0.05 ± 0.36D. In terms of safety, no patient lost two or more lines of CDVA. The authors concluded that SMILE had safety and efficacy similar to those of LASIK used for low-myopia. In another study, Autrata et al. [12] performed LASEK for low to moderate myopia with 20% alcohol inside the alcohol solution cone for 25-30 s. LASEK had faster vision recovery, milder pain, and a lower incidence of haze than PRK. At the 2-year follow-up visit, the safety and efficacy indexes were 1.04 and 0.98, respectively; 62% of the patients had a postoperative SE within ± 0.5D and 92% within ± 1.0D. Spadea et al. [13] used flap-preserved LASEK without alcohol to correct low to moderate myopia and obtained good outcomes with an efficacy index of 0.87 and a safety index of 1.25 after a follow-up of 60 months.

In addition to safety and efficacy, other considerations also should be considered when selecting surgical methods for patients. SMILE and LASEK have their own advantages and disadvantages. For example, SMILE maintains the integrity of the corneal epithelium and Bowman's layer and therefore has mild postoperative ocular discomfort, faster recovery, and is free of flap-related complications. However, SMILE requires 10–30 μm of additional base thickness in the corneal stroma, and thus more corneal tissue is removed using this technique than the application of excimer laser surgery. Furthermore, the cooperation of patients is critical during femtosecond laser scanning. In addition, the design of the parameters in the SMILE set for low myopia treatment should be more carefully determined since the outcomes are affected by the adjustment of the nomograms and the applied laser energy and femtosecond laser scanning quality [14]. LASEK is also a flapless procedure, which prevents flap-related complications. Compared with SMILE, less corneal tissue is removed in LASEK, and therefore it is more suitable for patients with relatively thin cornea. Additionally, the excimer laser machine has an eye-tracking system that is valuable in the process of excimer laser scanning and is hence more appropriate for patients with poor cooperation or large angle kappa. Previous studies have shown that postoperative pain of LASEK might be related to the time and concentration of alcohol used in the procedure [1, 12, 15]. In this study, 20% alcohol infiltration was applied for 12 s, after which the patients reported mild postoperative pain or discomfort. In addition, the medication time after LASEK is longer, and more frequent follow-up is needed.

We found that the increment of total corneal spherical aberrations (SA) and SA of the anterior cornea surface after SMILE were less than those after LASEK. Previous studies reported that the SA induced by SMILE was less while the coma error was higher than that of FS-LASIK [16, 17]. The increase of spherical aberration would augment the post-operative occurrence of halo in the dark. Zhu et al. [5] and Yu et al. [8] reported significantly lower HOAs and SA after SMILE than after LASEK for high myopia[5]or mild to moderate myopia [8], with no significant differences in the coma and trefoil aberrations between groups. In the current study, the vertical coma was significantly increased after SMILE, which was in line with the results of previous studies [18]. Lack of an eye-tracking system as well as involuntary Bell phenomenon in SMILE procedure may contribute to the increase of vertical coma.

In the clinic, many patients who complain of visual disturbances after refractive surgeries have visual acuities of 1.0 or better and refractions close to zero diopter, therefore, it is inadequate to assess their symptoms with conventional measures of acuity or refraction. Subjective visual quality after laser surgery should be given full attention. In this study, the main visual disturbances were starburst, halo, and vision fluctuation two years after the surgery. These symptoms were not reported to disturb patients' daily life except for inconvenience when driving at night postoperatively, which is consistent with the findings of Wei et al*. *[19]’s study which evaluated the subjective visual quality after SMILE for high myopic patients.

Our study has some limitations. First, we observed only the changes in the corneal aberrations, whereas the effect of total ocular aberrations on visual quality was not analyzed. Second, we included only patients with spherical equal to -3.00 D or less and astigmatism equal to 1.50 D or less, and thus the results could not be extrapolated to patients with spherical -3.00 D and astigmatism 1.50 D or more. Third, the sample size was small, and thus larger samples of observation with a longer follow-up period should be implemented in the future.

## Conclusions

In conclusion, SMILE and LASEK used for correction of low myopia provided good safety, stability, and visual quality. Nonetheless, the underlying mechanism leading to greater vertical coma in SMILE than in LASEK remains to be further explored.

## Data Availability

The data used during the current study are available from the corresponding author on reasonable request.

## Abbreviations

SMILE: Small-incision lenticule extraction
LASEK: Laser epithelial keratomileusis
UDVA: Uncorrected distance visual acuity
CDVA: Corrected distance visual acuity
IOP: Intraocular pressure
HOAs: Higher-order aberrations
SA: Spherical aberrations
SE: Spherical equivalent
CCT: Central corneal thickness
D: Diopters
AL: Axial length
RMS: Root mean square
Z3: -3, Vertical trefoil
Z3: -1, Vertical coma
Z3: 1, Horizontal coma
Z3: 3, Horizontal trefoil
